# Electromagnetic Acoustic Transducers for Robotic Nondestructive Inspection in Harsh Environments

**DOI:** 10.3390/s18010193

**Published:** 2018-01-11

**Authors:** Sungho Choi, Hwanjeong Cho, Matthew S. Lindsey, Cliff J. Lissenden

**Affiliations:** 1 School of Mechanical Engineering, Hanyang University, Seoul 04763, Korea; sunghochoi@hanyang.ac.kr; 2 Department of Engineering Science and Mechanics, The Pennsylvania State University, University Park, PA 16802, USA; huc146@psu.edu; 3 Applied Technology Group, Structural Integrity Associates, Inc., State College, PA 16801, USA; mlindsey@structint.com

**Keywords:** electromagnetic acoustic transducers (EMATs), robotic nondestructive inspection, harsh environments, elevated temperature, gamma radiation

## Abstract

Elevated temperature, gamma radiation, and geometric constraints inside dry storage casks for spent nuclear fuel represent a harsh environment for nondestructive inspection of the cask and require that the inspection be conducted with a robotic system. Electromagnetic acoustic transducers (EMATs) using non-contact ultrasonic transduction based on the Lorentz force to excite/receive ultrasonic waves are suited for use in the robotic inspection. Periodic permanent magnet EMATs that actuate/receive shear horizontal guided waves are developed for application to robotic nondestructive inspection of stress corrosion cracks in the heat affected zone of welds in stainless steel dry storage canisters. The EMAT’s components are carefully selected in consideration of the inspection environment, and tested under elevated temperature and gamma radiation doses up to 177 °C and 5920 krad, respectively, to evaluate the performance of the EMATs under realistic environmental conditions. The effect of gamma radiation is minimal, but the EMAT’s performance is affected by temperatures above 121 °C due to the low Curie temperature of the magnets. Different magnets are needed to operate at 177 °C. The EMAT’s capability to detect notches is also evaluated from B-scan measurements on 304 stainless steel welded plate containing surface-breaking notches.

## 1. Introduction

**Figure 1:**
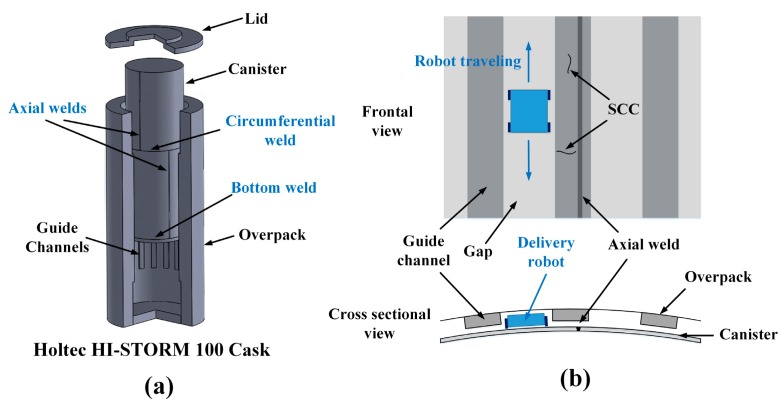
(**a**) A 3D cut-away view of the vertical-axis HI-STORM 100 cask; and (**b**) the frontal view from inside, and the cross-sectional view from above, showing geometric constraints between the canister and the overpack, and the limited accessibility to the axial weld when the weld is located at a guide channel.

There are many cases where nondestructive inspection (NDI) of structures, such as pipelines and containers, needs to occur in harsh environments, including elevated temperature and radioactivity in the nuclear power industry. An excellent example considered in this research is dry storage casks for spent nuclear fuel, shown in Figure 1a [1,2,3,4,5]. The dry storage cask consists of a welded austenitic stainless steel cylindrical canister that confines spent nuclear fuel and a concrete/steel overpack that shields and protects the canister. The canister is roughly 2 m in diameter and 5 m long [5]. Chloride-induced stress corrosion cracking (SCC) in the heat affected zone (HAZ) of full penetration welds in the canister is a potential aging mechanism of the canister [1,2,3,4,5,6,7,8,9,10], which should be inspected for in order to ensure the continued health and performance of the dry storage cask. However, the environment in the plenum between the canister and the overpack has both elevated temperature and gamma radiation that both gradually decrease over time in storage. Given the dynamic nature of the thermal and radiation fields predicted by numerical simulations, the robotic inspection system design criteria were conservatively selected to be 177 °C (350 °F) and 27 krad/h, respectively [1,5]. The harsh environment requires that the materials used in the sensors to detect SCC be carefully selected to maintain functionality throughout the inspections.

In addition to the harsh environment, geometric constraints inside the storage cask make the inspection of the canister even more challenging. The cask type shown in Figure 1 has vertical guide-channels attached to the steel cladding inside the overpack to maintain the concentricity between the canister and the overpack during handling and design service loading. The channels are nominally 50 mm deep and 150 mm wide, and their center-to-center spacing is 370 mm. The clearance between the channel and the canister surface is nominally 25 mm, but could range from 0 to 50 mm depending on the concentricity of the canister and overpack. Thus, this very small clearance with the uncertainly of the space makes it difficult to deploy the delivery robot for canister NDI under the channels. The obvious approach with the most certainty is that the delivery robot moves up and down in the air gaps between the guide channels, as shown in Figure 1b. Here, the delivery robot and inspection system must be designed considering the worst situation, and the minimum air gaps are nominally 50 mm deep and 214 mm wide, which provides a very limited space. Moreover, the guide channels restrict the delivery robot from approaching portions of the circumferential and bottom welds that are under the channels. Furthermore, if the axial weld is located at the channel, as shown in Figure 1b, it is completely inaccessible.

Some NDI techniques that could be used for assessment of SCC in a stainless steel canister have been identified in government reports [1,11,12,13]; visual testing (VT), eddy current testing (ECT), acoustic emission testing (AET), and ultrasonic testing (UT). VT and ECT are effective for detection of surface cracks. They have been well demonstrated and are frequently used in nuclear power plants. However, their applications are limited to only surface crack detection and cannot be used for volumetric characterization. Furthermore, the weld needs to be accessible to inspect it. AET is attractive for continuous global monitoring of SCC, but its sensitivity and reliability to SCC is a potential concern. Guided wave UT is effective not only for surface crack detection, but also for volumetric characterization of cracks. Lamb waves and shear horizontal (SH) waves are well known types of guided waves commonly used for NDI of plates and pipes [14]. Though Lamb waves (either symmetric (S) or anti-symmetric (A) modes) have been extensively used for defect characterization [15,16,17], they are not very effective for detection of cracks aligned in the wave propagation direction because they have particle motion that is entirely in the sagittal plane. On the other hand, SH waves essentially have wave motion with a three-dimensional profile; harmonic transverse displacement as a function of thickness. Thus, SH waves scatter and reflect from cracks not only perpendicular to the wave vector, but parallel to it [18]. It is also worth pointing out that SH waves are just as sensitive to cracks on the inside of the canister as they are on the outside, so that unexpected cracks on the inside of the canister will also be detected.

SH waves can be effectively excited and received by electromagnetic acoustic transducers (EMATs) or magnetostrictive transducers (MSTs). The advantages and disadvantages of each transducer when applied to robotic nondestructive inspection of spent nuclear fuel storage canisters were discussed in a previous publication [4]. MSTs rely on coupling through a frictional force between the transducer and the substrate, and provide much higher amplitude and signal-to-noise ratios (SNRs) than EMATs for SH waves. However, they require a large normal force to produce sufficient frictional forces and tend to be highly sensitive to surface conditions of the substrate including surface roughness and surface debris. These drawbacks of MSTs make practical application to robotic inspection of spent nuclear fuel storage canisters difficult. On the other hand, EMAT operation is based on noncontact transduction using the Lorentz force. Thus, there is no need to apply a normal force to the transducer and they are significantly less sensitive to surface debris and surface roughness than MSTs. For these reasons, EMATs were selected to generate/receive SH waves in the inspection of welded stainless steel canisters that store spent nuclear fuel. Note that the initial prototype EMATs used in the previous study [4] were not designed for elevated temperature and gamma radiation resistance.

This research develops EMATs that can operate inside a dry storage cask that has a challenging elevated temperature and gamma radiation environment. The next section describes the development of temperature and radiation tolerant EMATs and the selection of SH guided wave modes. Three different experiments are conducted to evaluate EMAT performance under elevated temperature and gamma radiation, and to assess their capability to detect notches. The experimental procedures and results are presented in Section 3, and are followed by the conclusions in Section 4.

## 2. Development of Temperature and Radiation Tolerant EMATs

### 2.1. Periodic Permanent Magnet EMATs

One feature of EMATs that makes them an attractive ultrasonic transducer is that they can generate and receive ultrasonic waves without a coupling medium owing to their non-contact transduction. The transduction of EMATs is based on the Lorentz force, which is the cross product of the alternating eddy current density driven by an electric coil and the static magnetic field near the surface of a conductive material [4,19]. Depending on the arrangement of the electric coil and permanent magnets, all types of ultrasonic waves, including bulk, Rayleigh, Lamb, and SH guided waves, can be excited and received [19]. Here we develop a looped coil and an array of magnets to actuate SH guided waves.

**Figure 2:**
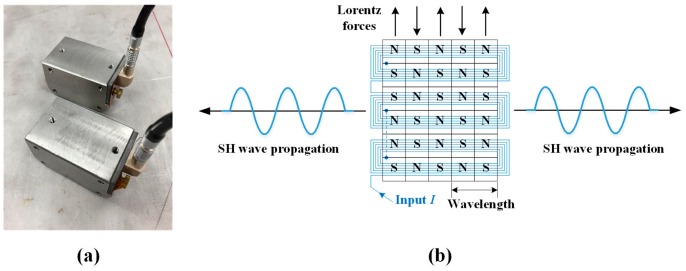
(**a**) Pair of periodic permanent magnet EMATs; and (**b**) the layout of permanent magnets and the electric coil to excite and receive SH waves.

A pair of prototype periodic permanent magnet EMATs were developed that have the layout of permanent magnets and an electric coil as shown in Figure 2. The transmitter and receiver are identical, each consisting of a looped electric coil, a periodic array of permanent magnets, a stainless steel housing, and peripheral components. The EMAT housing dimensions are 70 mm × 45 mm × 38 mm (length × width × height), which will fit between the overpack and the canister, but needs to be made more compact in the next design iteration to fit into the cargo bay of the delivery robot [5]. The periodicity of the magnet arrangement and the coils are arranged to the wavelength of the SH_0_ mode, 12.7 mm, at the predetermined frequency of 250 kHz. The same design is used for both EMATs so that each can perform send and receive functions by simply interchanging the coaxial cables.

### 2.2. SH Mode Selection

**Figure 3:**
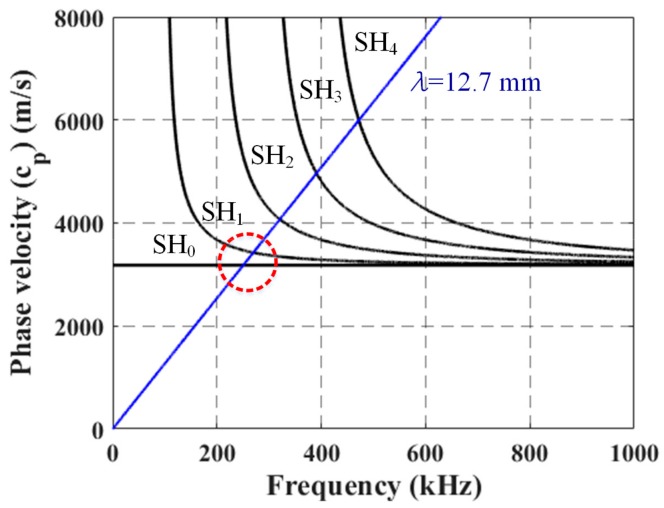
Phase velocity dispersion curves for the SH modes of a 15.9 mm thick stainless steel plate (black lines) with the activation line at a 12.7 mm wavelength for the designed periodic permanent magnet EMAT (blue line).

Considering the fact that some SH waves are dispersive, selection of optimal wave modes and their frequencies to be used is indispensable for successful NDI. Possible wave modes and their frequencies can be analyzed from the dispersion curves [14]. Phase velocity dispersion curves for the SH modes of a 15.9 mm thick stainless steel plate are shown in Figure 3 (canister wall thicknesses are typically 12.7 or 15.9 mm). Only the lowest five modes are shown here for simplicity. The fundamental SH_0_ mode is non-dispersive while the other modes are dispersive. The straight blue line represents the activation line, having a 12.7 mm wavelength, for the periodic permanent magnet EMAT, and the red circle roughly indicates a SH mode activation zone when a center frequency of 250 kHz is applied to the EMAT for wave excitation. The zone forms an area rather than just a point due to the source influence associated with the finite size of the EMAT and the finite frequency bandwidth of the tone-burst excitation sent to the EMAT [14]. This results in the EMATs activating both SH_0_ and SH_1_ modes simultaneously. The excitation frequency of 250 kHz with the 12.7 mm wavelength is selected as a compromise between defect sensitivity, EMAT size, and the presence of multiple SH modes nearby [4]. 

### 2.3. Temperature and Radiation Tolerant EMAT Components

The EMAT design requirements were conservatively set for the maximum operation temperature and the gamma radiation dose to be 177 °C (350 °F) and 27 krad/h, respectively. Table 1 shows the selected material for each component, and their maximum operational temperatures (provided by the vendors) and gamma radiation tolerances [20,21]. The rare earth magnets are made of neodymium alloy material, NdFeB. Their listed maximum operational temperature and Curie temperature are 80 °C and 310 °C, respectively. Therefore, they are not expected to tolerate the upper end of the design temperature range. This alloy was chosen because they are readily available, while higher Curie temperature magnets are made to order and have a longer lead time. The electrical coil is bonded flexible polyimide film with a circuit printed in the loop pattern shown in Figure 2b. Polyimide offers a maximum operational temperature of 200 °C and is known for its good resistance to gamma radiation [20]. The housing is machined from 316 grade stainless steel that has excellent elevated-temperature and gamma radiation tolerance. Likewise, Novalac-based high-temperature epoxy and polyether-ether-ketone (PEEK) have high temperature and gamma radiation tolerances [20,21].

## 3. Experiments and Discussions of Results

### 3.1. Elevated Temperature Tests

#### 3.1.1. Electric Coil Impedance Change with Temperature

There is usually a large impedance mismatch between an EMAT and the high-power electrical signal generator (or a signal acquisition device) that drives it [19]. This mismatch causes inefficient transfer of the electric power into ultrasound. Thus, it is essential to measure an EMAT’s impedance and construct an impedance matching network for optimal EMAT transduction efficiency. First, the resistance of the electric coil was measured up to 121 °C (250 °F). Note that the initial testing was not conducted up to 177 °C in recognition of the operating temperature of the magnets and limitations of the furnace that was available.

**Figure 4:**
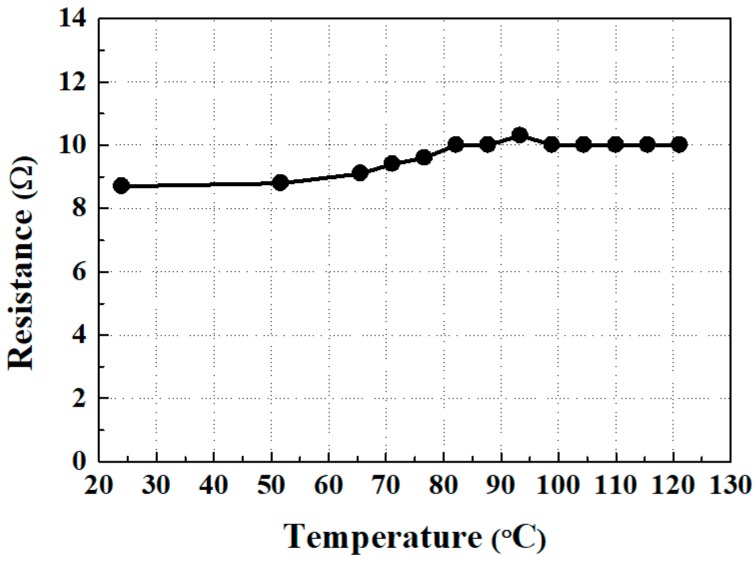
The electrical resistance change of the electric coil with the temperature.

The results of electric coil resistance change with the temperature are shown in Figure 4. The resistance is relatively constant over the full temperature range, and the values are in the range of 8.7–10.3 ohms. If it is determined that the resistance change has a significant effect on wave signal quality, it is possible that the impedance matching could be adjusted to a value that is optimized for the anticipated typical EMAT operating temperature. During this testing, a full impedance study was not necessary as the SNR was only slightly affected, leaving it unnecessary to adjust any matching electronics. Moreover, at each temperature interval of the resistance testing, a moderate shear force was applied to the coil to observe if any layer would become delaminated due to adhesive failure. No disbonds or damage was observed.

#### 3.1.2. EMAT Performance as a Function of Temperature

**Figure 5:**
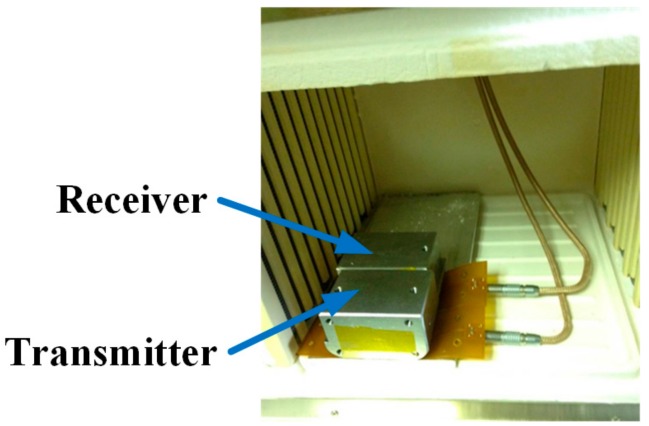
Temperature testing setup with EMATs placed side-by-side on an aluminum alloy plate.

The EMATs were tested in a laboratory furnace to evaluate their performance in elevated temperature environments up to 121 °C. The test setup is shown in Figure 5. The aluminum alloy test sample is 6.35 mm thick and 190.5 mm long, and the EMATs are placed side-by-side on the plate in pitch-catch operation. A portable high-power ultrasonic instrument (PowerBox H, Innerspec, Lynchburg, VA, USA) was used to provide a high-power electrical signal of five-cycle tone-burst with a 1200 V peak-to-peak voltage and a 250 kHz central frequency to the transmitter, and also to acquire ultrasonic signals from the receiver. During the acquisition of the signal, a 200~300 kHz band pass filter and a 12 dB pre-amplifier were utilized to enhance the SNR. The signals from the receiver were acquired after the recorded internal temperature of the laboratory furnace had stabilized for approximately 8 min.

**Figure 6:**
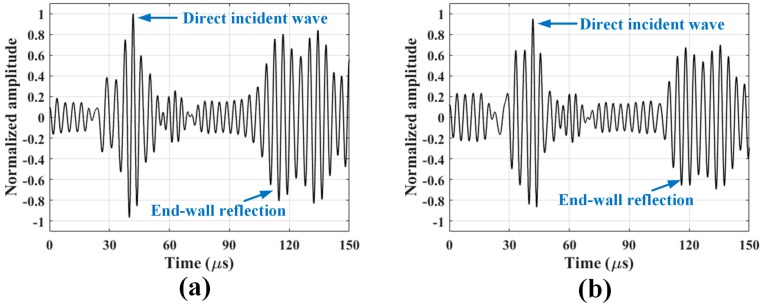
Normalized SH wave signals taken at (**a**) room temperature and (**b**) 121 °C, with respect to the peak amplitude of the direct incident wave at room temperature.

Typical SH wave signals taken at room temperature and 121 °C are shown in Figure 6, where the signals are normalized with respect to the peak amplitude of the direct incident wave at room temperature. Only the first two peaks, which correspond to the direct incident wave and the end-wall reflection, will be analyzed.

**Figure 7:**
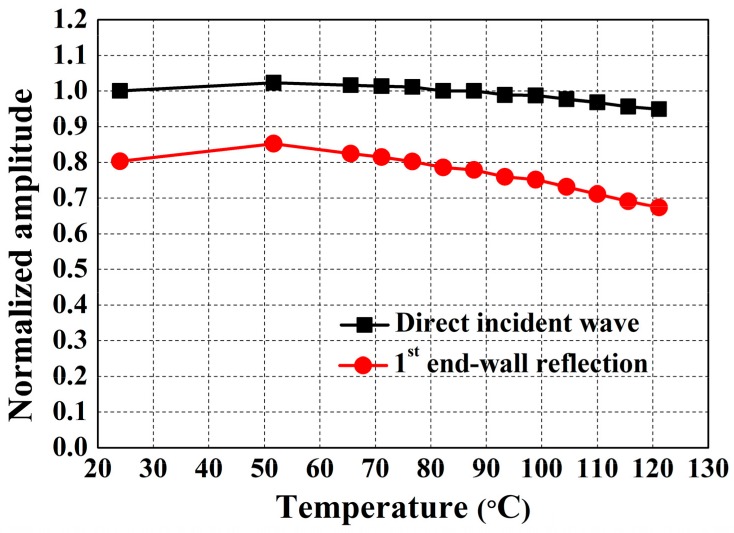
The normalized amplitudes of the direct incident wave and the first end-wall reflection as a function of the temperature.

The normalized amplitudes of the direct incident wave and the first end-wall reflection as a function of temperature are shown in Figure 7. First, the amplitude of the direct incident wave decreases by approximately 5% as the temperature increases to 121 °C. The 5% reduction can be conservatively regarded as a performance falling-off of the designed EMATs by the temperature environment because the propagation distance of the directed incident SH wave is very short and the temperature-dependent ultrasonic attenuation can be neglected. On the other hand, the end-wall reflection decreases by roughly 15%. This result means that based on the 5% EMAT’s falling-off described above, approximately 10% amplitude reduction arose by an increase in ultrasonic attenuation due to the temperature rise, which is reasonable because the propagation distance of the end-wall echo is much longer than the direct incident wave. Obviously, the results of the elevated temperature tests show promise of reliable EMAT operation up to 121 °C without significant adverse effects in the signal quality, even with the inevitable increase in attenuation.

**Figure 8:**
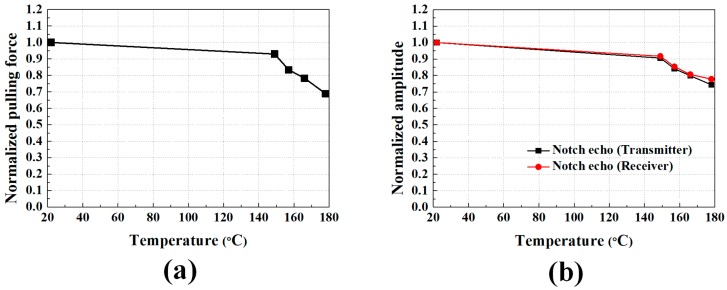
(**a**) The normalized pull force and (**b**) the normalized notch echo for neodymium magnets at room temperature after thermal exposure.

Satisfied that the magnets function well up to 121 °C, which is the temperature being used in some less conservative robotic inspection system designs for dry storage casks, we conducted a second stage of EMAT testing up to 177 °C. In these tests the magnetic force and EMAT signal strength were measured at room temperature after the magnets had been subjected to thermal exposures between 149 °C and 177 °C. Four magnets were exposed to sequentially higher temperatures up to 177 °C. After each 15-min temperature exposure, a magnet was placed between a steel block and rod. The pulling force was measured with a force gauge (SR-20, American Weigh Scales, Cumming, GA, USA) connected to the rod and results averaged together. Figure 8a shows the mean pull force as a function of temperature normalized with respect to the room temperature value (24 N). After exposure to 177 °C, the magnetic pull force was decreased by 31%. The same thermal exposures were applied to a 5 × 6 rectangular array of magnets and then the array was used as part of an EMAT to send or to receive SH waves in a 15.9 mm thick stainless steel plate with a surface-breaking semi-circular notch (depth was 5.3 mm). The notch was perpendicularly aligned to the wave vector and the distance to the receiving EMAT was set to 101.6 mm. The normalized amplitude of the notch echo is also shown in Figure 8b, and is seen to decrease by 26% and 23% for the magnets being used for transmitter and receiver, respectively. This result confirms that neodymium magnets lose a portion of magnetism when subject to heating above the listed operation temperature, which is 80 °C for the magnets, well below the Curie temperature. To ensure sufficiently long operation of EMATs up to 177 °C, higher thermal-grade magnets are required. For an example, commercially-available N40UH grade neodymium magnets can retain magnetic strength up to 180 °C [22], which needs to be considered during the next design iteration.

### 3.2. Gamma Radiation Tests

**Figure 9:**
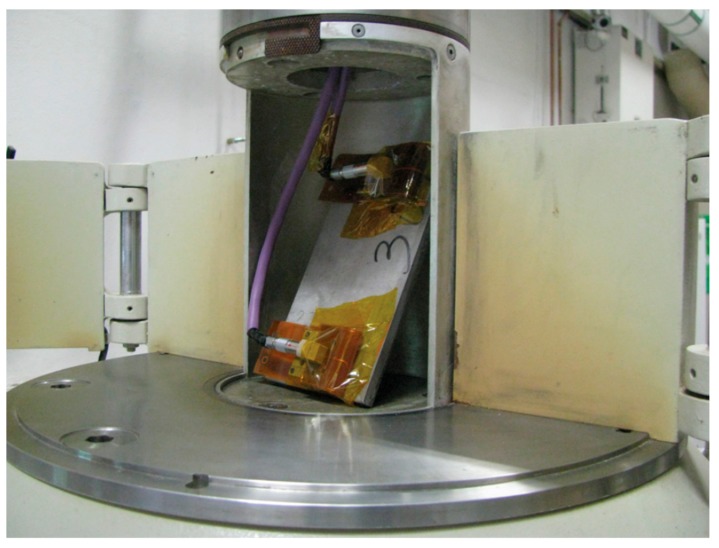
Gamma radiation test setup in the gamma cell where the transmitter and the receiver are separately attached to both ends of a 6.35 mm thick 304 stainless steel plate, and employ the through-transmission mode.

Accelerated gamma radiation tests were conducted on the primary EMAT components with no housing due the limited space inside the gamma cell at the Radiation Science and Engineering Center at Pennsylvania State University. The test setup is shown in Figure 9. The transmitter and the receiver are separated to prevent mutual attraction of the magnets during testing. They are attached to a 6.35 mm thick and 177.8 mm long 304 stainless steel plate and the through-transmission mode is used. The ultrasonic test system and excitation parameters are the same as in the elevated temperature tests. The gamma cell provides a dose rate of 370 krad/h from a cobalt-60 source. The test was conducted continuously for 16 h, which produces a total dose of 5920 krad. This cumulative dose is equivalent to 219 h of operation in a 27 krad/h environment.

**Figure 10:**
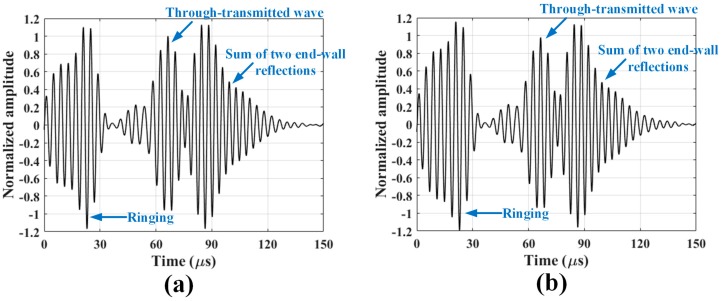
Normalized SH wave signals collected at (**a**) prior to exposure and (**b**) 16 h of gamma radiation exposure.

**Figure 11:**
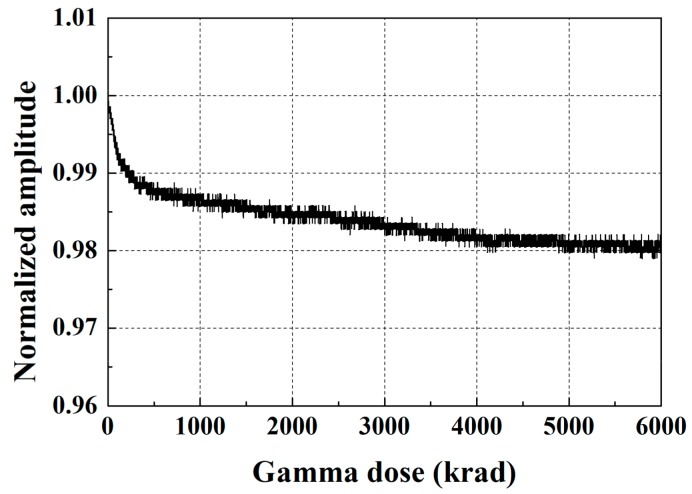
The normalized amplitudes of the incident wave as a function of gamma radiation dose.

Typical SH wave signals collected at the beginning of the testing and after 16 h of gamma radiation exposure are shown in Figure 10, where the signals are normalized with respect to the peak amplitude of the through-transmitted wave at the beginning of the testing. No meaningful changes in the peak amplitudes and waveforms, as well as no abnormal noise are observed. A slightly longer ringing can be observed in these signals compared to the temperature testing signals. This is attributed to the absence of a sensor housing and ringing within the magnets. The normalized amplitudes of the incident wave as a function of the gamma dose are shown in Figure 11. A very slight amplitude reduction occurs initially, and then the normalized amplitude converges to approximately 0.98. The 2% reduction is significantly smaller than the reduced performance by elevated temperatures. These experimental results show reliable EMAT operation up to a total dose of 5920 krad and beyond.

### 3.3. EMAT Capability to Detect Notches

**Figure 12:**
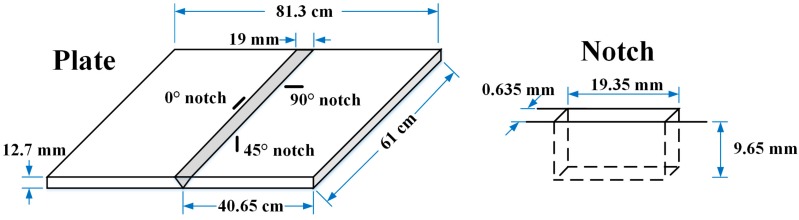
Plate and notch dimensions of a 12.7 mm thick 304 stainless steel welded plate having surface-breaking notches.

The EMATs were tested on a 12.7 mm thick 304 stainless steel welded plate having well-defined surface breaking notches near the HAZ to evaluate their notch sensitivity. Plate and notch dimensions are indicated in Figure 12. The plate contains three 75%-through-thickness notches implanted by electrical discharge machining having different orientations with respect to the weld line: 90°, 0°, and 45°. The length-depth ratio of the rectangular notches is 2:1. Of the three notches, the 90° and 0° notches are of primary interest to us due to the direction of the thermal residual stress driving force for SCC.

**Figure 13:**
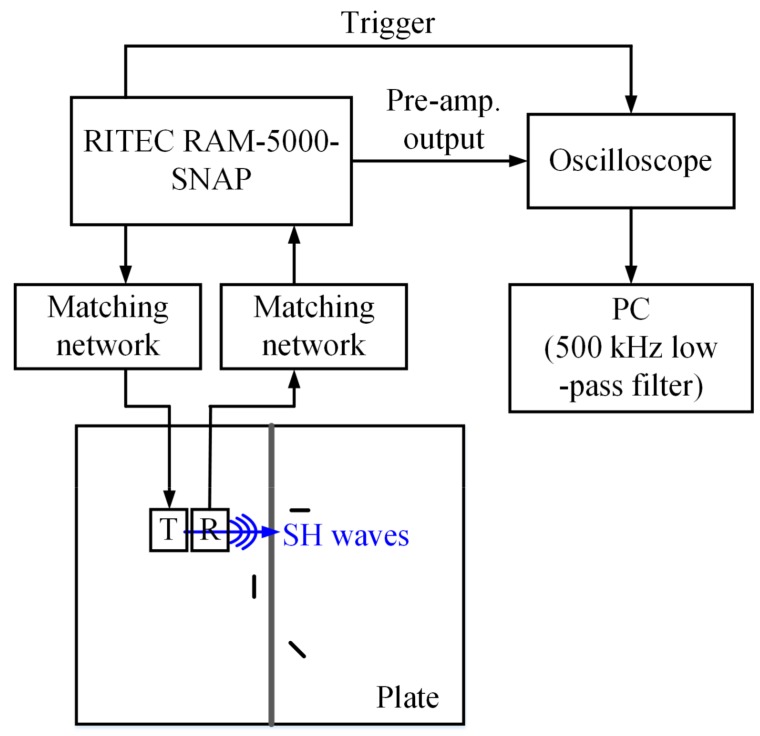
Schematic of the experimental setup to evaluate EMAT’s notch sensitivity.

A schematic of the experimental setup to evaluate the EMAT notch sensitivity is shown in Figure 13. The RAM-5000-SNAP (Ritec, Warwick, MA, USA) is used to produce a five-cycle tone-burst electric signal with a 1200 V peak-to-peak voltage and a 250 kHz central frequency. The electric signal passes through a matching network (TEM-128, Ritec), and then to the transmitter to generate SH waves on the plate. The acquired signal from the receiver goes through another matching network (REMP-128, Ritec) and a 30 dB pre-amplifier in the RAM-5000-SNAP. Then, the pre-amplified signal is displayed on an oscilloscope (MSO 2014, Tektronix, Beaverton, OR, USA) and recorded. For post-processing, a 500 kHz digital low-pass filter is applied to the recorded signal to eliminate high-frequency noise.

**Figure 14:**
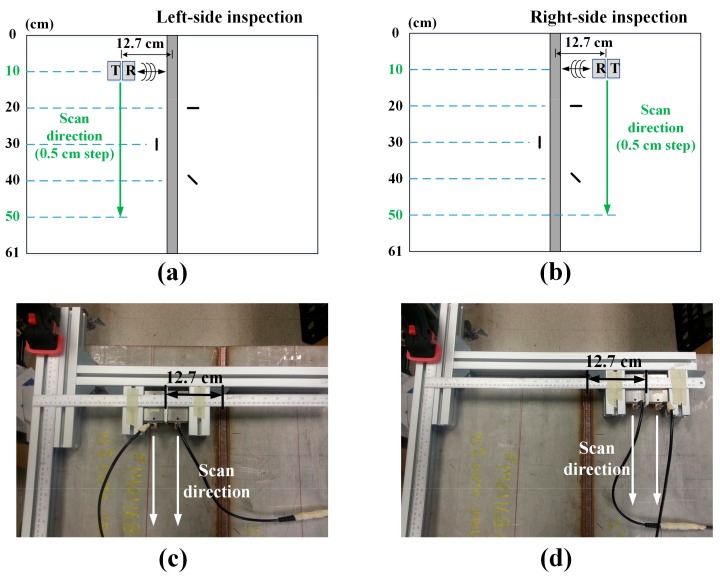
Notched plate inspection schemes and corresponding experimental setups: (**a**,**c**) left-side inspection; and (**b**,**d**) right-side inspection.

The notched plate inspection schemes and corresponding experimental setups are shown in Figure 14. Both the left and right sides of the weld line are inspected. The pair of EMATs is scanned in the direction parallel to the weld line maintaining a fixed distance between EMATs and the weld line to obtain B-scan images. The scan was performed from 10 to 50 cm with a step of 0.5 cm, based on the upper edge of the plate. Here, the locations of the 90° and 0° notches are 20 and 30 cm, respectively.

**Figure 15:**
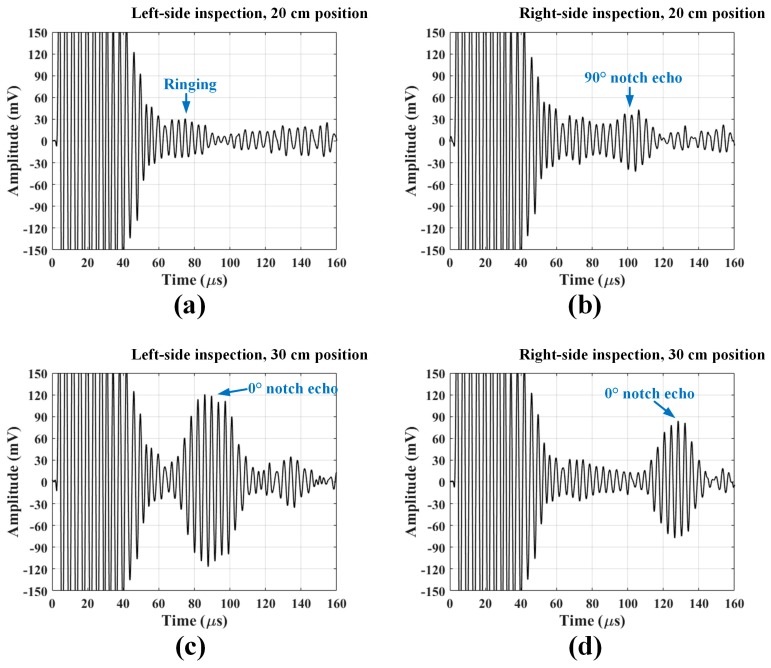
The waveform signals taken when the EMAT pair is located at (**a**,**b**) 20 cm position; and (**c**,**d**) 30 cm position.

Figure 15a,b show the A-scans taken when the EMAT pair is located at the 20 cm position in the left and right-side inspections, respectively. In the left-side inspection, the 90° notch echo is not detected due to the following two phenomena: the scattering from the notch parallel to the wave vector is, in general, small and the small scattered waves may be severely attenuated as they pass through the weld. Meanwhile, the right-side signal shows a clear notch echo arriving at about 100 μs. This result demonstrates that it is possible to detect the parallel notch with the EMATs if the SH waves do not pass through the weld. The waveform signals related to 0° notch detection measured at 30 cm position are shown in Figure 15c,d. The reflected waves from the notch are clearly observed at both left and right-side inspections. The 0° notch perpendicular to the wave vector reflected much more energy than the parallel notch.

**Figure 16:**
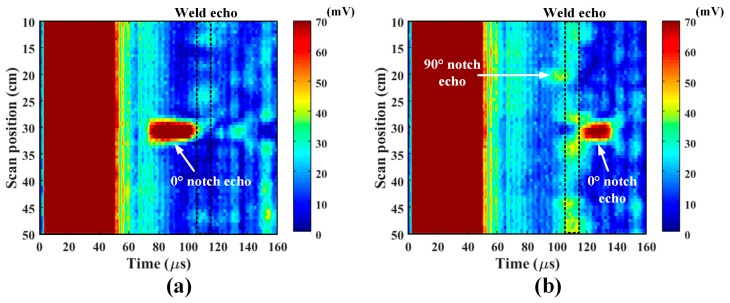
B-scan images of the notch plate at (**a**) left-side inspection; and (**b**) right-side inspection.

B-scan images of the notch plate are shown in Figure 16, where the Hilbert transformation was applied to each A-scan signal to obtain the envelope of the signal. The images display the absolute magnitudes of the enveloped signals in a color map. The waves arriving at approximately 110 μs are weld echoes. Though the echo amplitude varies with the scan position due to the non-uniformity of the weld quality, its arrival time is consistent. The echo from the 0° notch is clearly shown before the weld echo in the left-side inspection, and after the weld echo in the right-side inspection. The 90° notch echo is also shown before the weld echo in the right-side inspection. However, the 45° notch was not detected since the notch deflection traveled off at a 45° angle and was not received by the EMAT. Overall, the experimental results show that the designed EMATs have good sensitivity to both notches, although the sensitivity to the parallel notch is certainly lower than that for the perpendicular notch.

## 4. Conclusions

Temperature and radiation-tolerant EMATs have been developed for robotic nondestructive inspection of stress corrosion cracks in the weld HAZ of stainless steel dry storage canisters. Periodic permanent magnet EMATs that actuate and receive SH waves were fabricated, where the EMAT components were carefully selected in consideration of the inspection environment which includes elevated temperature and gamma radiation. The designed EMATs were tested under elevated temperature and gamma radiation doses, up to 177 °C and 5920 krad, respectively. Gamma radiation had no significant effect for this dose. While no significant degradation of the EMAT’s performance was observed up to 121 °C, both the magnetic pull force and the SH wave amplitude are significantly reduced due to thermal exposures from 121–177 °C. Clearly, neodymium magnets having a higher Curie temperature are needed for service temperatures above 121 °C. Additionally, the EMAT’s capability to detect notches was evaluated from B-scan measurements on a 304 stainless steel welded plate containing surface-breaking notches. The EMATs provided acceptable detectability for notches oriented perpendicular or parallel to the wave vector when the notch tip was not in the weld itself. Thus, the experimental results confirm that the EMATs are suitable for robotic inspection of dry storage casks. Future work will reduce the size of the housing to fit into the cargo bay of the robotic cars and evaluate higher Curie temperature magnets to enable inspections between 121 and 177 °C.

## Figures and Tables

**Table 1:** EMAT components selected for their harsh environmental resistances.

Components	Material	Function	Elevated Temperature (from the Vendors)	Gamma Radiation [20,21]
Rare earth magnets	NdFeB	Provide a static magnetic field	Operational temp.: 80 °C Curie temp.: 310 °C	-
Electrical coil substrate	Polyimide	Fixes coil position, which creates eddy currents	200 °C	1 × 10^6^ krad
Housing	Stainless steel 316	Encloses magnets and coil	1400 °C	>1 × 10^6^ krad
Epoxy adhesive	Novalac-based high temperature epoxy	Secures magnets in housing	260 °C	8.8 × 10^4^ krad
Coil-fixation tape	Polyimide	Secures coil to magnets	200 °C	1 × 10^6^ krad
Wear-resistant tape	PEEK	Protects both substrate and coil from wear damage and sets liftoff	204 °C	1 × 10^6^ krad
